# Extracellular Vesicle-Induced Classical Complement Activation Leads to Retinal Endothelial Cell Damage via MAC Deposition

**DOI:** 10.3390/ijms21051693

**Published:** 2020-03-01

**Authors:** Chao Huang, Kiera P. Fisher, Sandra S. Hammer, Julia V. Busik

**Affiliations:** Department of Physiology, Michigan State University, East Lansing, MI 48824, USA; chaohuang@uabmc.edu (C.H.); fishe247@msu.edu (K.P.F.); shammer@msu.edu (S.S.H.)

**Keywords:** diabetic retinopathy, extracellular vesicles, complement system, extracellular vesicles, retinal vascular damage, human retinal endothelial cells (HRECs), membrane attack complex (MAC)

## Abstract

Several studies have suggested that there is a link between membrane attack complex (MAC) deposition in the retina and the progression of diabetic retinopathy (DR). Our recent investigation demonstrated that circulating IgG-laden extracellular vesicles contribute to an increase in retinal vascular permeability in DR through activation of the complement system. However, the mechanism through which extracellular vesicle-induced complement activation contributes to retinal vascular cytolytic damage in DR is not well understood. In this study, we demonstrate that IgG-laden extracellular vesicles in rat plasma activate the classical complement pathway, and in vitro Streptozotocin (STZ)-induced rat diabetic plasma results in MAC deposition and cytolytic damage in human retinal endothelial cells (HRECs). Moreover, removal of the plasma extracellular vesicles reduced the MAC deposition and abrogated cytolytic damage seen in HRECs. Together, the results of this study demonstrate that complement activation by IgG-laden extracellular vesicles in plasma could lead to MAC deposition and contribute to endothelium damage and progression of DR.

## 1. Introduction

Extensive experimental and clinical evidence supports the link between complement system activation and the pathogenesis of diabetic vascular complications, including diabetic retinopathy (DR) and atherosclerosis [1,2]. The complement system is an effector for adaptive and innate immunity that is activated via three enzymatic cascades known as the classical, the mannose-binding lectin (MBL), and alternative pathways [3,4]. All three pathways eventually converge at the level of complement component 3 (C3) and thereafter share a common sequence of C5 convertase formation and generation of the membrane attack complex (MAC) (C5b-9). The formation of MAC results from the binding of C5b to complement proteins C6, C7, C8, and multiple molecules of C9. Once formed, MAC complex leads to osmotic imbalance and ultimately lysis of pathogens or cells. To prevent unintended damage to the host tissue by activated complement cascade, several complement regulatory proteins (CD55, CD46, and CD59) are anchored on the plasma membrane via a glyphosphatidylinositol. These regulatory proteins protect host cells from complement-induced self-cell damage [3]. However, aberrant complement activation and impairment of complement regulatory proteins in pathological conditions can lead to MAC formation on host cells [3,4]. An increased level of MAC deposition found in the eyes of patients with DR when compared to eyes from non-diabetic subjects [5], is suggested to be the result of both the reduced levels of complement regulatory proteins and continued activation of the alternative pathway [6]. Moreover, glycosylation-induced impairment of functional activity of complement regulatory protein CD59 may also contribute to complement activation in pathologies such as diabetes [7]. Increased MAC deposition in diabetic tissues has been implicated in the release of growth factors that promote abnormal cell proliferation in the vascular wall of vessels, thus contributing to the development of vascular proliferative disease [8]. However, whether complement activation and MAC deposition participate in retinal endothelium damage remains unclear.

Intriguingly, complement components have been found to associate with extracellular vesicles, such as exosomes, in circulation [9,10]. Extracellular vesicles are small vesicles measuring 40–200 nm in diameter [11], and are found in most biological fluids including blood, urine, cerebrospinal fluid, and ascites [12]. Importantly, extracellular vesicles released from parental cells carry biological information such as nucleotides, proteins, and lipids that exert various effects on target cells. In fact, it has been suggested that extracellular vesicles may serve as a novel cell-cell communication method due to the heterogeneity of the cargo they carry from cell to cell [13]. Recently, we have reported that plasma extracellular vesicles activate the classical complement pathway, and may contribute to MAC-induced retinal vascular permeability in diabetes [14]. However, whether MAC induces cellular damage in the diabetic retinal vasculature remains unknown. In this study, we investigated the physiological mechanism in which diabetic plasma extracellular vesicles induce complement activation leading to MAC deposition and cellular impairment in human retinal endothelial cells (HRECs).

## 2. Results

### 2.1. Immunoglobulins Are Associated with Extracellular Vesicles in Rat Plasma

**Figure 1:**
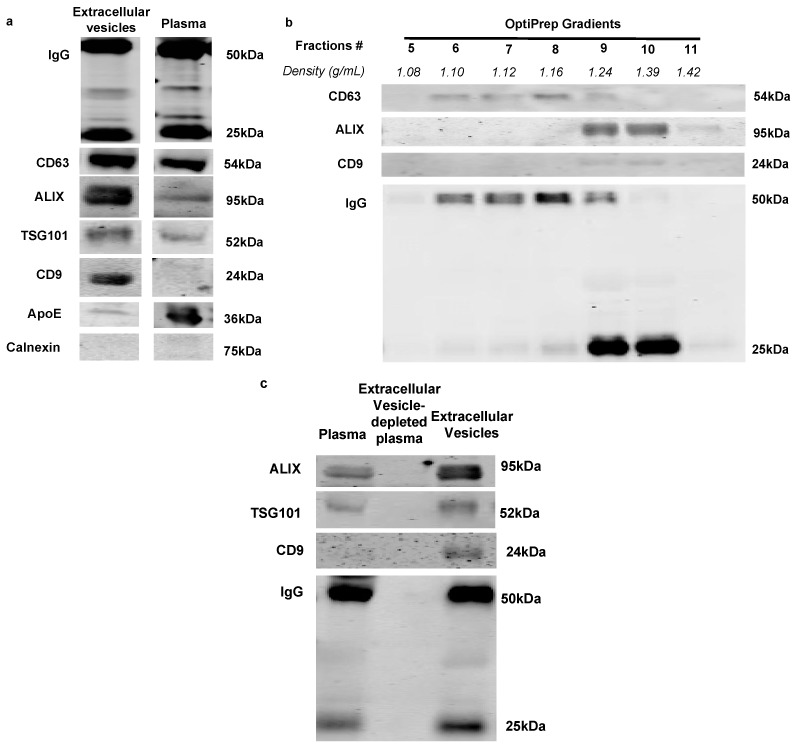
Characterization and specificity of immunoglobulin binding to extracellular vesicles. (**a**) Western blot analysis of rat plasma and ultracentrifugation isolated extracellular vesicles under equal loading volume condition; isolated rat plasma extracellular vesicles showed an enrichment of extracellular vesicle markers (CD63, ALIX TSG101, CD9) compared to whole plasma. The specificity of extracellular vesicle isolation is confirmed using lipoprotein marker (ApoE) and endoplasmic reticulum (ER) marker Calnexin. (**b**) Western blot analysis of extracellular vesicles with OptiPrep density gradient separation post-ultracentrifugation isolation. Extracellular vesicle markers CD63, ALIX and CD9, as well as Immunoglobulin, are present in fractions with 1.10–1.39 g/mL density (fractions 6 to 10). (**c**) Immunoglobulin is removed in extracellular vesicle-depleted plasma (middle lane) via combined ExoQuick and ultracentrifugation methods. All the results were repeated in three different experiments and cropped images in (**a**) between plasma and extracellular vesicles were from the same membrane and uncropped images can be found in Supplementary Figures.

Previously, we have demonstrated that immunoglobulins are associated with the extracellular vesicles isolated via the ExoQuick method in mouse plasma [13]. The ExoQuick method provides a fast and efficient way to isolate extracellular vesicles; however, it is not specific for extracellular vesicles and can precipitate a wide range of extracellular vesicles and proteins that could potentially affect the results of this study. In this study, sufficient plasma volume from the rat model allowed us to precipitate extracellular vesicles via ultracentrifugation method, allowing for better purity and more homogeneous extracellular vesicle isolation. We observed that rat extracellular vesicles isolated by ultracentrifugation showed an enrichment of classical extracellular vesicle markers (CD63, ALIX, TSG101 and CD9) compared to total plasma (Figure 1a). In agreement with our previous study, immunoglobulins were also enriched in rat plasma extracellular vesicles (Figure 1a) [13]. The purity of the isolation was further confirmed by a reduction of LDL lipoproteins (ApoE) and Calnexin markers (Figure 1a, bottom) in the extracellular vesicle preparation. OptiPrep density gradient was used post sequential ultracentrifugation to demonstrate the presence of IgG in extracellular vesicle fractions. We found that fractions 6 to 10 were positive for extracellular vesicle markers (CD63 and ALIX), where IgG were also present (Figure 1b). After depleting extracellular vesicles from plasma by combined ExoQuick and ultracentrifugation isolations, we found an associated depletion in IgG levels in the rat plasma (Figure 1c, middle lane). These data are consistent with our previously published results [13], which found that in rat plasma, immunoglobulins are associated with extracellular vesicles.

### 2.2. IgG-Laden Extracellular Vesicles Activate the Classical Complement Protein C1 Complex

**Figure 2:**
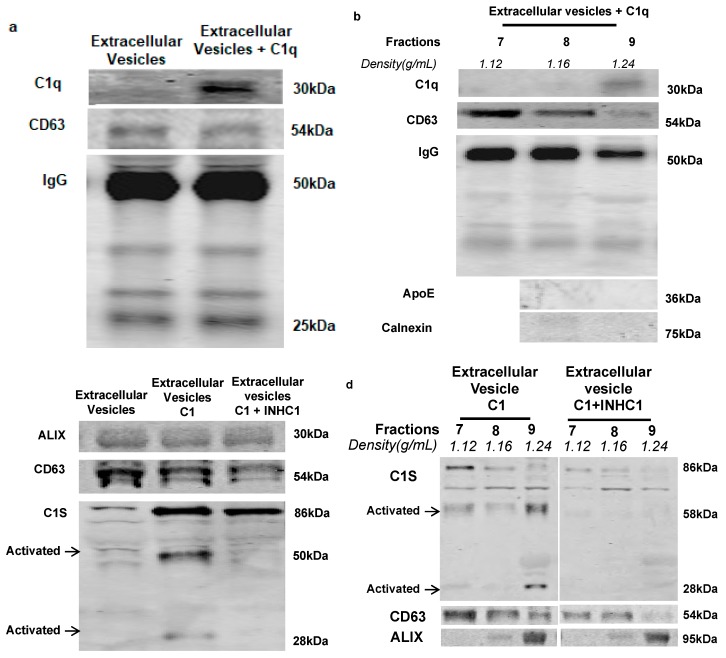
Western blot analysis of complement activation with rat plasma extracellular vesicle isolated via ultracentrifugation and purified by OptiPrep density gradient. (**a**) Binding of human complement protein, C1q, to ultracentrifugation isolated rat plasma extracellular vesicles. (**b**) C1q specifically bound to rat plasma extracellular vesicles with density at 1.24 g/mL (fraction 9). This fraction was negative for ApoE and Calnexin. (**c**) C1 activation assay showed that C1 was activated by ultracentrifugation-isolated rat plasma extracellular vesicles (middle-lane) and C1 activity was inhibited in the presence of C1 inhibitor (INHC1) (right-lane). (**d**) Incubating C1 complex with rat plasma extracellular vesicles followed by fractionation using OptiPrep density gradient showed that C1 complex bound to extracellular vesicle at 1.24 g/mL density (fraction 9) and activated serine protease subcomponent C1s (left), and the activity of the C1s was inhibited by C1 inhibitor (INHC1) (right). All of the experiments were repeated at least three times.

Immunoglobulin is a potent activator of the classical complement pathway. C1q is the first classical complement protein that associates with immunoglobulin [14]. To examine whether IgG-laden rat plasma extracellular vesicles activate the classical complement pathway, we performed a C1q binding assay [15]. The rat plasma extracellular vesicles for this experiment were isolated by ultracentrifugation in order to reduce the non-extracellular vesicle contamination. Results of the C1q binding assay showed that purified human C1q protein (CompTech, A099) binds to IgG-laden rat plasma extracellular vesicles (Figure 2a). OptiPrep density gradient centrifugation post C1q binding assay then demonstrated that human C1q binds to rat plasma extracellular vesicles in fraction 9 (Figure 2b), in the absence of lipoprotein (ApoE) and ER marker (Calnexin) (Figure 2b, bottom).

Once C1q binds to the classical complement activator, auto-activation of complement serine protease C1r occurs. This in turn cleaves and activates another serine protease C1s in the C1 complex [2]. To investigate if C1q binding to extracellular vesicles activates the C1 complex, we performed the C1 activation assay [15]. Rat plasma extracellular vesicles isolated by ultracentrifugation were incubated with purified human C1 complex (CompTech, A098) and activation of C1 was measured by a cleaved form of C1s. Rat plasma extracellular vesicles activate C1 (Figure 2c, middle lane), and C1 activity was reduced in the presence of human C1 inhibitor (C1-INH) (CompTech, A140) (Figure 2c, right lane). OptiPrep density gradient showed that C1 activation occurred in the same fraction (fraction 9) that was also positive for exosomal markers (CD63 and ALIX) (Figure 2d, left lane). These results suggest that IgG-laden rat extracellular vesicles bind to C1q and activate the classical complement protein, C1, in plasma.

### 2.3. Quantification of Extracellular Vesicles in Rat Control and Diabetic Artery Plasma

**Figure 3:**
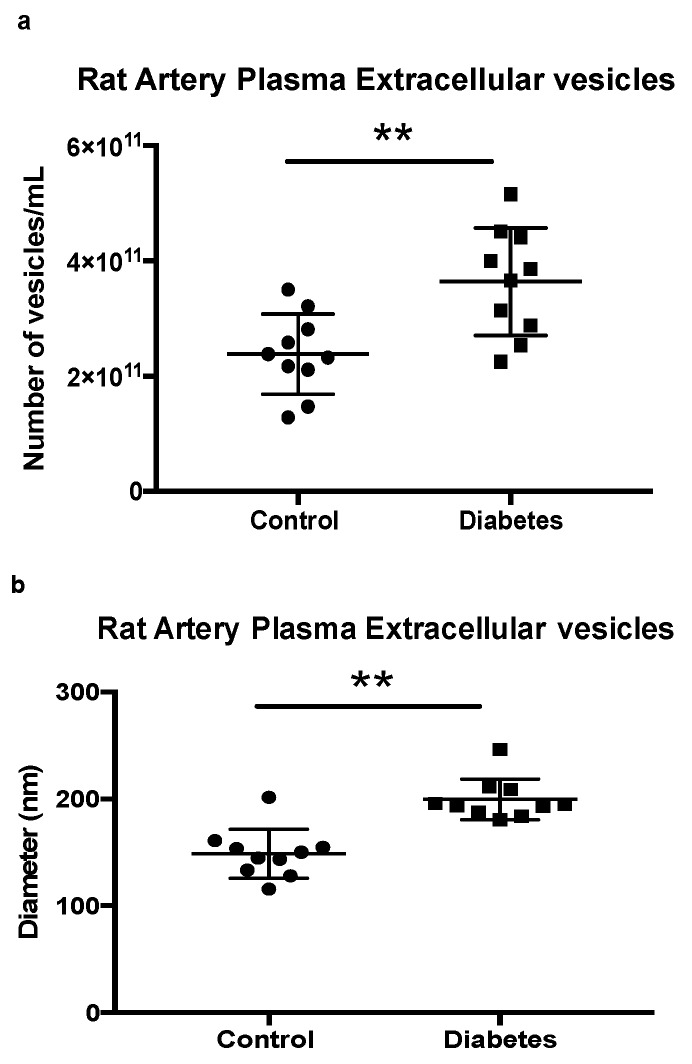

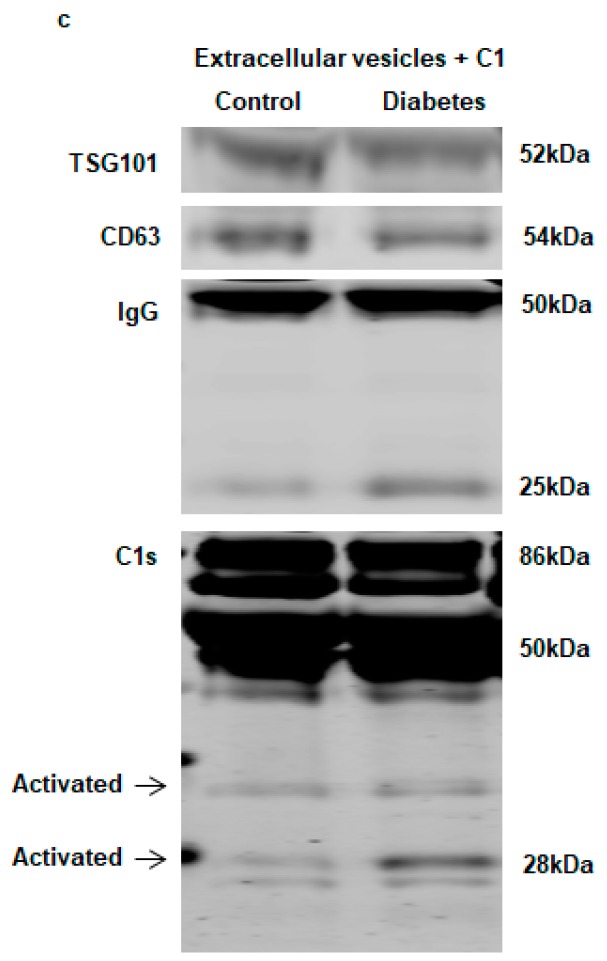
Comparison of rat artery plasma extracellular vesicles isolated via ExoQuick from control and STZ-induced diabetic (7 weeks) rats. (**a**) The number of vesicles was increased significantly in diabetic (7 weeks) rat plasma than in control. The number of vesicles isolated from plasma of control rats (circles) and STZ-induced diabetic rats (squares) was measured through the use of SLS (kilocounts per second). (**b**) Dynamic light scattering (DLS) measurement showed an increase of diameter in diabetic vesicles (squares) compared with control (circles). *n* = 10 for control and diabetes, ** *p* < 0.05. (**c**) Western blot of artery plasma vesicles isolated through the use of ExoQuick from control and STZ-induced diabetic (7 weeks) rats. Under condition of equal volume loading, extracellular vesicle markers (TSG 101 and CD63) show no difference between combined control and diabetic plasma vesicles; however, an increased amount of IgG was observed in diabetic rat plasma, but not with control rat plasma. Moreover, diabetic rat artery plasma vesicles had higher C1 activity than control vesicles.

Previously, we have demonstrated a novel extracellular vesicle quantification method by using combined dynamic and static light scattering (DLS and SLS) assays with Zetasizer Nano NZ (Malvern Instruments Ltd., Malvern, UK) [13]. To reduce the technical variability caused by the extracellular vesicle isolation procedure, we used ExoQuick isolation method instead of ultracentrifugation for these measurements. Quantification of isolated extracellular vesicles with Zetasizer showed that STZ- induced (seven weeks) diabetic rats have a higher number of extracellular vesicles in the artery plasma when compared to non-diabetic rat plasma (Figure 3a). This is consistent with our previous observation using plasma from STZ-induced diabetic mice [13]. Interestingly, the diameter of the extracellular vesicles found in diabetic artery blood also increased when compared to extracellular vesicles from non-diabetic controls (Figure 3b). However, a Western blot analysis of rat plasma extracellular vesicles demonstrated that there was no change in extracellular vesicle markers (TSG 101 and CD63) between combined control or combined diabetic plasma (Figure 3c). The IgG level, however, was higher in diabetic rat plasma extracellular vesicles than in control plasma extracellular vesicles (Figure 3c). Moreover, C1 activation assay demonstrated that there was a greater C1 activation in diabetic rat extracellular vesicles than in control rat extracellular vesicles (Figure 3c). These data demonstrated that diabetic rats showed an increase of total number of extracellular vesicles in arterial circulation, but no change between control and diabetic TSG101 and/or CD63 positive extracellular vesicles. Moreover, the physical properties of the circulating extracellular vesicles, as measured by vesicle diameter, have changed due to the onset of diabetes when compared to controls. Additionally, a higher C1 activity in diabetic extracellular vesicles may have contributed by the increased number of IgG-laden extracellular vesicles in the diabetic rat artery.

### 2.4. Diabetic Rat Plasma Extracellular Vesicles Contribute to HREC Cytotoxicity via MAC Deposition

**Figure 4:**
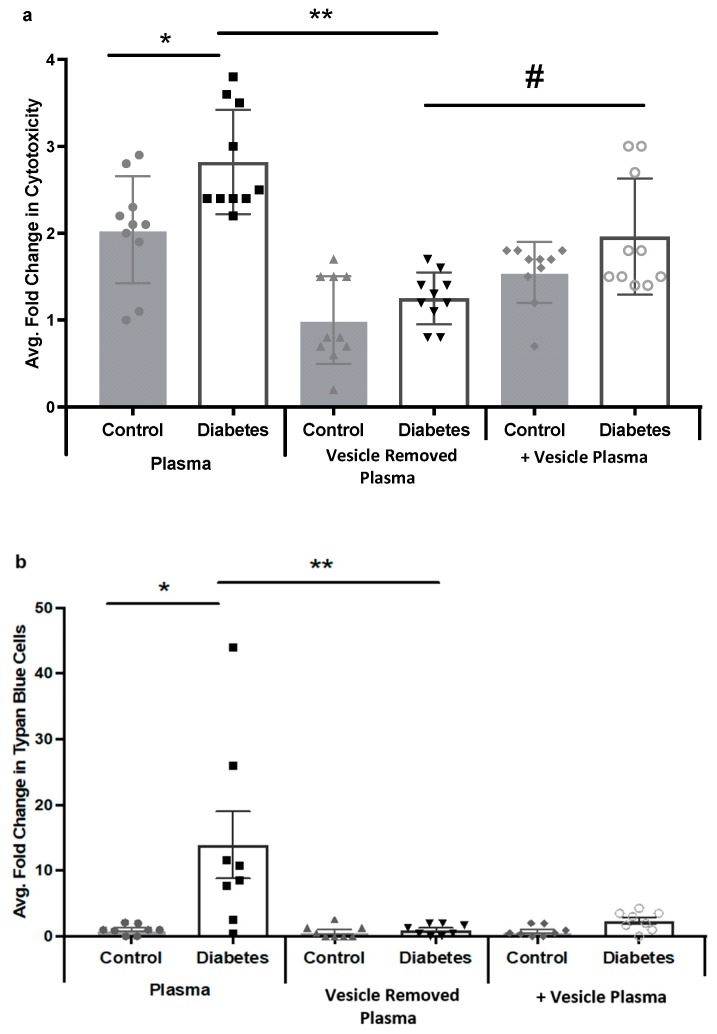

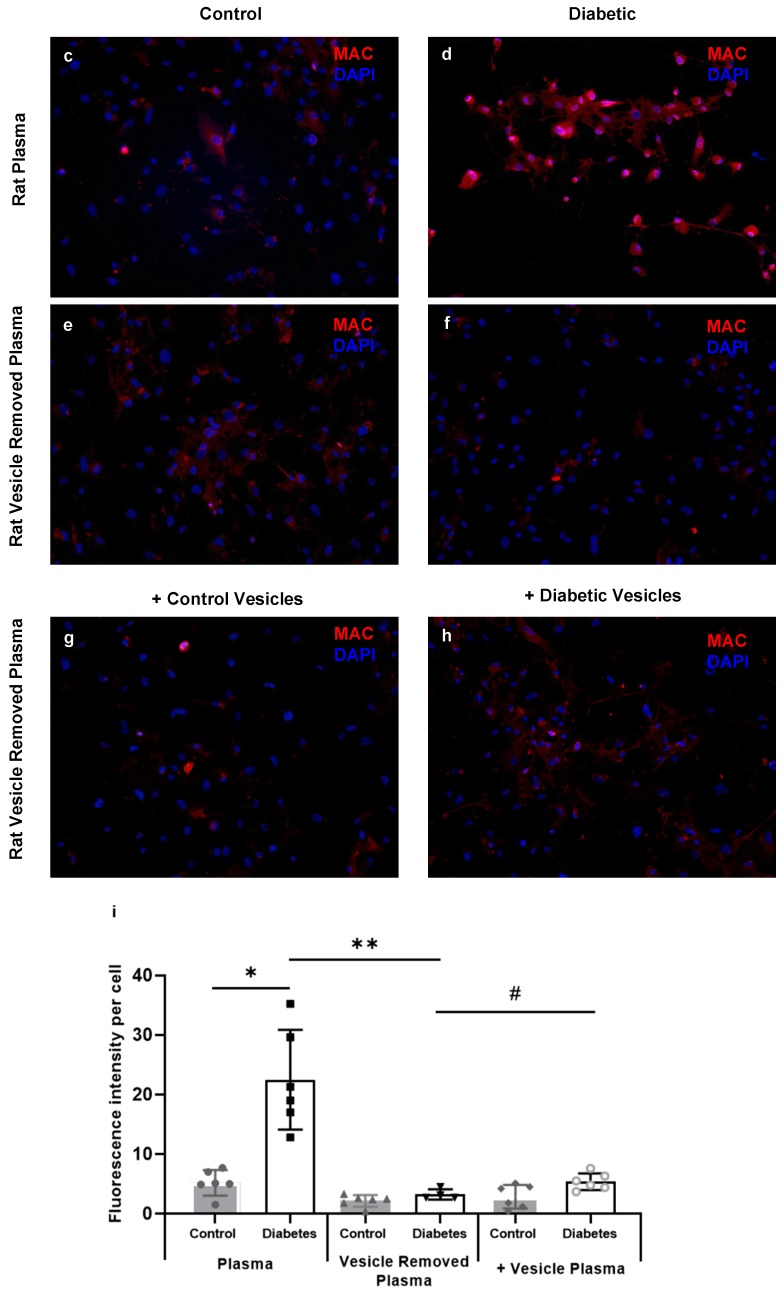
Diabetic rat plasma induced cytotoxicity and cell death in human retinal endothelial cells (HRECs). STZ-induced diabetic (seven weeks) rat plasma was collected from tail artery and extracellular were removed via ExoQuick combined with ultracentrifugation method. (**a**) With LDH Assay; after 6 h, (*) diabetic rat plasma (white bar) treated human retinal endothelial cells (HRECs) showed a significant increase of cytotoxicity compared to control plasma treated HRECs (grey bar). (**) When extracellular vesicles were removed from diabetic plasma (white bar), cytotoxicity was reduced compared with diabetic plasma treated HRECs (white bar). (#) After adding diabetic extracellular vesicles back into the vesicle-removed plasma condition, cytotoxicity showed a significant increase when compared with the diabetic vesicle-removed condition (white bars). LDH concentration from no cell conditions were subtracted from each condition before calculation of cytotoxicity. (**b**) Trypan Blue staining of detached HRECs showed similar results as LDH measurement. Experiments were performed in triplicates following the assay manufacturer’s recommendation, and results are presented as mean ± SD. Control plasma treated HRECs were used for normalization to other conditions in LDH and trypan blue assays. * *p* < 0.05, ** *p* < 0.01. (**c**–**h**) HRECs were treated with rat plasma in different conditions for 3 h, and rat MAC (red) deposition and cellular nuclei (DAPI) were measured by immunocytochemistry. Diabetic rat plasma-induced MAC deposition in HRECs. (**d**). MAC deposition was greatly reduced in HRECs treated with vesicle-removed diabetic rat plasma (**f**). Once diabetic extracellular vesicles were added back into the plasma, MAC deposition was observed again (**h**), while all the controls remain unchanged (**c**, **e** and **g**). (**i**) Fluorescence quantification of the MAC deposition in HRECs, fluorescence intensity and the number DAPI were measured by MetaMorph software. All images were taken and analyzed with 10× magnification.

During the terminal complement cascade, complement proteins C5b, C6, C7, and C8 were assembled on the cell membrane into C5b-8 complex that binds C9 proteins and then intercalates into the plasma membrane, creating the MAC. To examine if plasma extracellular vesicle-activated complement cascade leads to MAC formation and cell lysis, we treated HREC with 20% of control or diabetic rat plasma in the presence or absence of extracellular vesicles. Cytotoxicity was measured by Lactate Dehydrogenase (LDH) assay, trypan blue exclusion assay, and MAC formation was examined by using immunocytochemistry. STZ-induced diabetic (7 weeks) and control rat tail-artery whole blood was collected into EDTA coated tubes, and plasma was isolated by centrifugation. To remove plasma extracellular vesicles, we combined ExoQuick with ultracentrifugation methods to maximize the extracellular vesicle depletion. LDH Assay was used to quantify the cytotoxicity of the conditioned media. We first determined LDH levels in control and diabetic rat plasma before and after exosome removal. We found that rat plasma contained LDH and there was an increased amount of LDH in the diabetic plasma. Interestingly, LDH levels were reduced to the background level with exosomes removed in both control and diabetic plasma, and were increased again when exosomes were added back (Figure S1). To account for the LDH present in the plasma containing exosomes, controls sister plates with no HREC were included in each experiment and were subtracted from the experimental plates. The LDH assay showed a significant increase in HRECs cytotoxicity in the cells treated with diabetic rat plasma compared to the cells treated with control rat plasma (Figure 4a). The removal of extracellular vesicles from the diabetic rat plasma significantly reduced cytotoxicity (Figure 4a). Interestingly, addition of diabetic extracellular vesicles back into the vesicle-removed plasma resulted in increased cytotoxicity when compared with the diabetic vesicle-removed condition (Figure 4a). The trypan blue assay showed similar results, with a substantial increase in cell death in HRECs treated with diabetic rat plasma, compared to control plasma treated cells (Figure 4b). This phenotype was reversed by the removal of extracellular vesicles from diabetic plasma (Figure 4b). To investigate whether the complement terminal cascade was involved in the cytotoxicity, rat plasma treated HRECs were stained with anti-MAC (C5b-9) antibody (Abcam, ab55811). A large number of HRECs treated with diabetic rat plasma detached from the culture plates within 6 h after treatment. In 3 h, remaining diabetic plasma treated HRECs became round, granular and stained positive with MAC (Figure 4d). In contrast, when HRECs were treated with diabetic vesicle-removed plasma the cells remained viable, attached, and negative on MAC staining (Figure 4f). Moreover, when diabetic extracellular vesicles were added back into the diabetic vesicle-removed plasma condition, MAC deposition was observed in HRECs, but this deposition was less than in the diabetic rat plasma condition (Figure 4h). HRECs treated with rat control plasma remained viable, and showed negative MAC staining (Figure 4c,e,g). Overall, these data suggest that arterial diabetic plasma extracellular vesicles contribute to MAC-deposition and cytolytic damage in HRECs.

### 2.5. Scanning Electron Microscopy (SEM) Analysis of Plasma and Extracellular Vesicles

**Figure 5:**
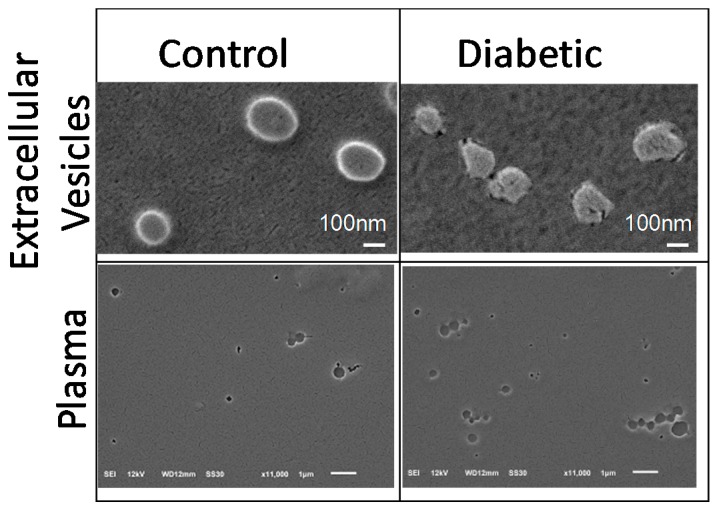
Scanning electron microscopy (SEM) of rat plasma and ultracentrifugation isolated extracellular vesicles. Extracellular vesicles (top) or plasma (bottom) were isolated from (left) control or (right) diabetic rats after seven weeks of STZ diabetes. Cholesterol crystals were not visible, via SEM, in isolated extracellular vesicle or plasma samples acquired from either control or diabetic rats.

As cholesterol crystals were recently shown to have plasma cytotoxicity [16]. SEM under non-dehydrating conditions was performed on the samples used in HREC treatment experiments (Figure 5). SEM confirmed the presence of extracellular vesicles of ~100–200 nm diameter in both control and diabetic extracellular vesicle preparations. Blood plasma contained the particles ranging from ~5–700 nm diameter. There were no crystalline structures observed in blood plasma or extracellular vesicles isolated from control or diabetic animals. Non-hydrating SEM is not the ideal method to preserve the structures, thus these experiments were performed to rule out the role of cholesterol crystals, rather than to quantify and characterize the extracellular vesicles.

### 2.6. MAC Deposition in the Retinal Vascular of Diabetic Rats

**Figure 6:**
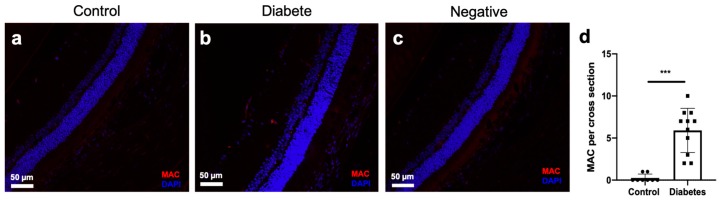
Membrane attack complex (MAC) deposition in the retina of rats with seven-weeks duration of STZ-induced diabetes. Retinal sections from a control rat (**a**) showed less MAC immunostaining (red fluorescence) than in a diabetic rat (**b**). (**c**) MAC negative staining of the diabetic rat retina section. Deposition and cellular nuclei (DAPI) staining in blue fluorescence. (**d**) Counts of the number of MAC deposits per retinal cross-section, *** *p* < 0.001, *n* = 7–11.

Seven weeks after the STZ-induction of the diabetic rats, immuno-stained rat retina showed an increase of MAC deposition when compared to controls (Figure 6). These experiments demonstrated that MAC deposition occurred in the eyes of diabetic rats.

## 3. Discussion

Under normal physiological conditions, low-level complement activation and non-cytolytic MAC deposition are thought to be beneficial, serving as a mechanism to remove opsonized cellular debris and pathogens [17] Complement activation and choriocapillaris loss in early AMD: Implications for pathophysiology and therapy, as well as maintaining the eyes immune privilege. However, under disease conditions such as those present in DR, continued complement activation and reduced level of complement regulatory proteins are suggested to be associated with increased MAC formation in the retinal endothelium lumen [6]. Moreover, irreversible MAC deposition on endothelial cells leads to the release of mitogenic factors [18], further supporting the rationale that complement activation is involved in the advancement of DR. Furthermore, circulating plasma extracellular vesicles have been shown to play a role in complement pathway activation leading to retinal vascular damage in vivo [13]. No studies, however, have investigated the role that diabetic extracellular vesicles play in causing cytolytic damage to the endothelium in vitro. In this study, we demonstrate that diabetic extracellular vesicles induce complement activation and MAC deposition, which contributes to retinal endothelial cell damage. The results of this study suggest that extracellular vesicle-induced complement activation followed by MAC deposition could provide a novel mechanism for increased retinal vascular permeability and DR pathogenesis.

Previously, we have demonstrated that immunoglobulins are associated with extracellular vesicles in circulation and activate classical complement protein C1 in both a human and mouse model [13]. In the current study, we demonstrated similar findings using rat plasma extracellular vesicles isolated by the ultracentrifugation method. These data suggest that activation of classical complement by IgG-laden extracellular vesicles occurs across species and is not dependent on the isolation method. Consistent with our previous finding, C1 activation occurs in a specific extracellular vesicle fraction (Figure 2d), which further supports the rationale that a sub-population of extracellular vesicles may favor complement activation. Ultracentrifugation method isolated rat plasma extracellular vesicle samples had a reduced level of lipoproteins when compared to the plasma (Figure 1a). This once again demonstrated that ultracentrifugation is the method of choice for a higher purity of extracellular vesicle isolation [19] and it, therefore, is ideal for extracellular vesicle characterization. However, the ultracentrifugation method is not quantitative due to the inherent variability of this multi-step process and is thus not suitable for comparing samples between control and diabetic groups. The ExoQuick isolation method was consequently the alternative method of choice to compare the number of vesicles between control and diabetic extracellular vesicles.

Ogata N. et al. demonstrated that an increase of extracellular vesicles in diabetic plasma could contribute to the acceleration of DR progression [20,21]. We have recently reported, using a novel extracellular vesicle quantification method in mice, that diabetes causes an increase in the number of circulating extracellular vesicles found in venous blood [13]. In agreement with this finding, our present study demonstrates that the number of extracellular vesicles is also increased in the arterial blood of STZ-induced diabetic rats when compared to controls (Figure 3a). However, in contrast with our previously reported venous samples, the diameter of the extracellular vesicles increased more in diabetic arterial circulation than in control. Additionally, arterial extracellular vesicles contain a wider range of CD63 positive vesicles in the Opti-prep density gradient (Figure 1b) and a higher value of polydispersity (data not shown) than our previously reported venous extracellular vesicles. Due to these findings, we speculate that extracellular vesicle populations may change as they pass from arterial to venous vascular beds and shift the density of the extracellular vesicles. Interestingly, extracellular vesicle markers TSG101 and CD63 showed no difference between control and diabetes in arterial blood (Figure 3c) while the total number of extracellular vesicles increased in diabetes. This suggests that TSG101 and CD63 positive extracellular vesicles may not contribute to the increased number of vesicles in diabetic atrial blood. It is also possible that ExoQuick isolated plasma extracellular vesicles contain other vesicles such as lipoproteins [22], which could contribute to the vesicle differences seen between the control and diabetic condition. Due to the rat plasma containing these varied populations, OptiPrep gradient fractionation is needed after the ExoQuick method to isolate the IgG-laden extracellular vesicles and observe their effect in future studies. Our current study demonstrated that the IgG level was higher in isolated diabetic arterial extracellular vesicles than in control extracellular vesicles. In agreement with the previous study [13], C1 activation at the vesicle fractions had a higher density (1.24 g/mL and above), where ALIX and CD9 expression were high, but low on CD63 expression. Heterogeneity of the extracellular vesicles on marker expression and the functional level was reported by several groups and is an active area of investigation [11,12,13]. Importantly, in this study, we demonstrated for the first time that diabetic extracellular vesicles have higher C1 activity than control extracellular vesicles.

In physiological states, a low level of complement activation in the eye is tightly regulated and MAC deposition on the surface of cells is normally rapidly removed. Endothelial cells are targeted continuously by activated complement cascade, and MAC deposition is rapidly eliminated via endocytosis, protecting the cells from cytolytic destruction [23]. A similar mechanism was reported to also occur in retinal pigment epithelial cells (RPEs) [24]. Moreover, it has been suggested that, in vitro, MAC-induced mitogenesis contributes to focal tissue repair or pathological cell proliferation [25]. In the present study, we observed that MAC deposition occurred in the retina of STZ-induced diabetic rats (Figure 6); this is consistent with previous studies in the eyes of diabetic patients and animal models [6,7]. Besides, we show evidence suggesting that diabetic rat plasma induces robust MAC deposition and cytolytic damage in HRECs, but not with control plasma (Figure 4). We reason that in the early stages of the DR, increased non-cytolytic retinal vascular MAC deposition contributes to signal focal tissue repair. As the disease advances, sustained complement activation accompanied by complement regulatory proteins impairment [6] and deficient of retinal tissue repair processes [26] heighten the MAC deposition, and this may contribute to cytolytic damage and increased retinal permeability. Previously, Kim D et al. demonstrated that complement regulatory proteins are homologous restricted and are less effective on complement targets from different species [27]. Moreover, gal-(alpha 1-3)-gal epitopes on endothelial cells have a high affinity to natural antibodies, such as IgG from different species and favor classical complement-induced xeno-organ rejection [28]. These reports suggest that in our study, surface-expressed complement regulatory proteins on HRECs might be less effective in protecting cells from rat extracellular vesicle-induced complement activation. Furthermore, HRECs might be sensitized by rat extracellular vesicle-associated and/or extracellular vesicle-unassociated immunoglobulins, which favor classical complement activation. Additionally, in diabetic plasma, hyperglycemia is usually accompanied by an elevated level of inflammatory cytokines and chemokines; this pro-inflammatory environment, coupled with the extracellular vesicles′ ability to carry proteins, nucleotides, and lipids makes extracellular vesicles more likely to facilitate cellular damage. Thus, we speculate that in diabetic rat plasma there are factors other than extracellular vesicle-induced complement deposition that may contribute to HREC damage. Interestingly, when we deplete extracellular vesicles from the diabetic plasma, complement-induced MAC deposition and cytolysis of HRECs were prevented. This suggests that plasma extracellular vesicles, in part, contribute to complement-dependent retinal cellular injury. However, the addition of diabetic plasma extracellular vesicles back into the diabetic extracellular vesicle-free plasma environment did not result in a full recovery of the cytotoxic effect, suggesting that extracellular vesicle isolation methods employed in our study may have inhibited proteins that are important for cellular cytotoxicity. These results highlight the importance of continued investigation and development of extracellular vesicle isolation techniques.

Recently, complement activation by cholesterol crystals was shown to contribute to vascular damage in human atherosclerosis and animal models [16,29]. To rule out the role of cholesterol crystals in HREC activation and MAC deposition, we performed SEM under non-dehydrating conditions to preserve potential cholesterol crystals. We observed the extracellular vesicles of expected 100–200 nm diameter in both control and diabetic extracellular vesicle preparations, and lipid particles ranging from 5~700 nm in blood plasma. There were no cholesterol crystals in control or diabetic plasma, or extracellular vesicle preparations used for HREC treatments.

In summary, our study demonstrates that diabetic rat plasma extracellular vesicles activate a greater level of classical complement protein C1 than control plasma extracellular vesicles. Activation of the complement cascade, in turn, contributes to MAC deposition and cytolytic damage of the retinal endothelial cells in diabetes. These findings may provide a novel mechanism that could contribute to endothelial cell dysfunction and the advancement of DR pathogenesis. Future research directions may also be highlighted.

## 4. Materials and Methods

### 4.1. Animal Studies

All animal procedures were in compliance with the National Institutes of Health (NIH) Guide for the Care and Use of Laboratory Animals and were approved and monitored by IACUC at Michigan State University (AUF Busik08/17-151-00, approval date: 28/08/2017–28/08/2020). Male Sprague-Dawley rats (237–283 g) were made diabetics with a single intraperitoneal injection of streptozotocin (STZ) (65 mg/kg) (Sigma Aldrich) dissolved in 100 mM citric acid (pH = 4.5) [30]. Body weight and blood glucose concentration were monitored biweekly and blood glucose concentration was maintained in the 20 mmol/L range. Rats with 7 weeks since onset of diabetes were used in this study.

### 4.2. Cell Culture

Primary human retinal endothelial cells (HRECs) were prepared from postmortem tissue obtained from National Disease Research Interchange, Philadelphia, PA, and EverSight Midwest Eye-Banks, Ann Arbor, MI. Primary HREC were isolated and cultured as previously described [31]. Passages 3–6 were used in the experiments at 80–90% confluence in 10% FBS media before treatments.

### 4.3. Western Blot

Western blot analysis was performed as previously described, with the following antibodies at dilution 1:1000: anti-CD63, anti-CD9 and anti-TSG101 (SBI, Cat.NO.EXOAB-CD63A-1, EXOAB-CD9A-1 and EXOAB-TSG101-1), anti-ALIX (AbCam, Cat. No. ab117600), anti-C1q (CompTech, Cat. No. A200), anti-C1s (Quidel, Cat. No. A302). IRDye Donkey anti-rabbit or anti-goat was used as secondary antibodies (Rockland, Cat. No. 611-731-127) (LI-COR, Cat. No. 925-32213). Immuno-reactive bands were visualized by using the Odyssey digital imaging program.

### 4.4. Blood Sample Collection

Blood was collected from inferior vena cava or from tail artery of the animal into tubes with EDTA (SARSTEDT microvette-300). Plasma was harvested via centrifugation and either used immediately or aliquoted into 1.5 mL micro-tubes and stored in −80 °C.

### 4.5. Extracellular Vesicle Isolation

Sequential ultracentrifugation method for extracellular vesicle extraction was conducted as previously reported [32]. In short, 0.5 mL of rat plasma was mixed with an equal volume of PBS, and then a series of low speed centrifugations of the supernatant were conducted to remove cellular debris. The supernatant was then filtered via a 0.22 µm filter, and extracellular vesicles were precipitated by spinning at 100,000 g for 2 h at 4 °C in a SORVALL M120SE Micro-Ultracentrifuge (S55S-1155 Rotor, SORVALL). The extracellular vesicle pellet was washed by re-suspension with PBS and spun down at 100,000 g before further analysis. ExoQuick (SBI, EXOQ5A) isolation was conducted based on manufacturer’s protocol.

### 4.6. Extracellular Quantification

Extracellular vesicle quantification was conducted based on previously reported procedures by combining dynamic light scattering (DLS) and static light scattering (SLS) technologies via Zetasizer Nano NZ (Malvern Instruments Ltd., Malvern, UK) [13].

### 4.7. OptiPrep Density Gradient Extracellular Vesicle Purification

Discontinuous iodixanol gradient was used to further purify the extracellular vesicle solution. Purification of extracellular vesicles by OptiPrep density gradient was done as previously described [15].

### 4.8. C1q Binding Assay

C1q binding assay was performed based on previously published procedures with modifications [33,34]. Isolated rat plasma extracellular vesicles (from 0.5 mL plasma) were re-suspended in 100 uL HEPES buffer (150 mM NaCl, 2 mM CaCl_2_, 20 mM HEPES, pH 7.0) and incubated with 2 ug of C1q (CompTech. A099) for 30 min at 37 °C. After the incubation, extracellular vesicles were isolated via ultracentrifugation, purified by OptiPrep density gradient, and then analyzed by Western blot.

### 4.9. C1 Activation Assay

The ability of extracellular vesicles to induce C1 activation was measured using an in vitro assay as previously published with modifications [15]. Extracellular vesicles were isolated from 0.5 mL of rat plasma, re-suspended in C1 activation assay buffer (50 nM triethanolamine-HCL, 145 mM NaCl, 1 mM CaCl_2_, pH 7.4) and incubated with C1 complex (0.25 uM) (CompTech, A098) in the presence or absence of C1 inhibitor (INHC1) (CompTech, A140) for 90 min at 37 °C. The reaction mixtures were incubated on ice for 10 min, submitted to OptiPrep density gradient purification to isolate extracellular vesicles, and the activation of C1s was analyzed by Western blot.

### 4.10. LDH Assay

Cytotoxicity of the cells was quantified using an LDH assay kit, following the manufacturer’s protocol (Abcam, ab102526). First, 105 HRECs/100 µL were plated onto a 0.1% gelatin coated 96-well plate, incubated at 37 °C for 48 h, and then treated with 20% of control or diabetic rat plasma at 37 °C for 6 h. Additional conditions were created to determine the contribution of extracellular vesicles vs. other plasma components to cell cytotoxicity. Vesicle-removed control or diabetic plasma, and vesicle-removed control or diabetic plasma with control or diabetic extracellular vesicles added back in were used. After the treatment with plasma, the media was collected into a 1.5 mL Eppendorf tube and spun down at 10,000 g for 15 min at 4 °C. Supernatants (50 µL) were transferred into a 96 well plate and a mixed detection kit reagent (50 µL) was added to each well along with the NADH standard. The absorbance (450 nm) was taken on a micro-plate reader in a kinetic mode every 3 min for 30 min at 37 °C. LDH activity of the samples was calculated: LDH activity (milli-units/mL) = amount of NADH (nmol)/(Reaction Time × Sample Volume) × Dilution Factor. LDH concentration from no cell conditions (Figure S1) were subtracted from each condition before calculation of cytotoxicity.

### 4.11. Immunocytochemistry

Immunocytochemistry was performed as previously described using anti-MAC (C5b-9) antibody (Abcam, ab55811) at 1:100 in PBS with 1.5% BSA overnight at 4 °C, followed by chicken anti-rabbit Alexa Fluor 594 secondary antibody (1:500) and DAPI (Sigma-Aldrich, St.Louis, MO, USA) nuclei counterstaining. The slides were analyzed using Nikon TE2000 microscope equipped with Photometric Cool-SNAP HQ2 camera. All images were taken with matched exposure time for experimental and control slides by using the MetaMorph imaging software (Molecular Devices, Downington, PA, USA).

### 4.12. Electron Microscopy

The blood plasma and extracellular vesicles were prepared for scanning electron microscopy (SEM) as previously described, omitting standard ethanol dehydration and critical point drying steps [16]. The samples were smeared on cover slips, mounted on SEM stubs, fixed with osmium tetroxide vapor and gold coated in an EMSCOPE SC500 sputter coater (Emscope, Ashford, UK) followed by examination using a JEOL SEM (model JEOL-6610LV, JEOL Ltd., Tokyo, Japan).

### 4.13. Statistics

A Student paired t-test was used to analyze data with two groups. In experiments with multiple group comparisons, one-way ANOVA with post-hoc analysis by Tukey’s range test (GraphPad Prim 7, GraphPad Software, San Diego, CA, USA) was used. All values are expressed as mean ± Standard Deviation. *p*-values below 0.05 were considered significant.

## Supplementary Materials

Supplementary materials can be found at https://www.mdpi.com/1422-0067/21/5/1693/s1.

## Abbreviations

DR	Diabetic Retinopathy
MAC	Membrane Attack Complex
HREC	Human Retinal Endothelial Cell
RPE	Retinal Pigment Epithelial Cell
