# Supplementary material for: Extracellular Vesicle-Induced Classical Complement Activation Leads to Retinal Endothelial Cell Damage via MAC Deposition
Source: Int J Mol Sci. 2020 Mar 1;21(5):1693. doi: 10.3390/ijms21051693 (PMC7084203; doi:10.3390/ijms21051693)
Supplement: Supplementary file 1 [file ijms-21-01693-s001.pdf]

Supplement Figure 1

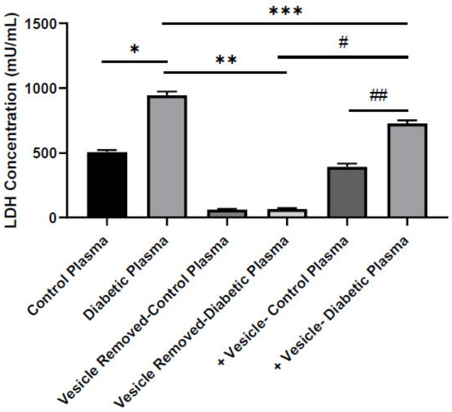

Supplement Figure 1. LDH concentration of control and diabetic plasma. Diabetic rat plasma contained higher concentration of LDH compared with control rat plasma. The LDH level decreased when extracellular vesicles were removed and it was restored after the extracellular vesicles were added back.
